# Receipt of COVID-19 Vaccine During Pregnancy and Preterm or Small-for-Gestational-Age at Birth — Eight Integrated Health Care Organizations, United States, December 15, 2020–July 22, 2021

**DOI:** 10.15585/mmwr.mm7101e1

**Published:** 2022-01-07

**Authors:** Heather S. Lipkind, Gabriela Vazquez-Benitez, Malini DeSilva, Kimberly K. Vesco, Christina Ackerman-Banks, Jingyi Zhu, Thomas G. Boyce, Matthew F. Daley, Candace C. Fuller, Darios Getahun, Stephanie A. Irving, Lisa A. Jackson, Joshua T.B. Williams, Ousseny Zerbo, Michael M. McNeil, Christine K. Olson, Eric Weintraub, Elyse O. Kharbanda

**Affiliations:** ^1^Yale University, New Haven, Connecticut; ^2^HealthPartners Institute, Minneapolis, Minnesota; ^3^Center for Health Research, Kaiser Permanente Northwest, Portland Oregon; ^4^Marshfield Clinic, Marshfield, Wisconsin; ^5^Kaiser Colorado, Denver, Colorado; ^6^Harvard Pilgrim Health Care Institute, Boston, Massachusetts; ^7^Kaiser Southern California, Pasadena, California; ^8^Kaiser Permanente Washington Health Research Institute, Seattle, Washington; ^9^Denver Health, Denver, Colorado; ^10^Vaccine Study Center, Kaiser Permanente Northern California, Oakland, California; ^11^CDC.

COVID-19 vaccines are recommended during pregnancy to prevent severe maternal morbidity and adverse birth outcomes; however, vaccination coverage among pregnant women has been low (*1*). Concerns among pregnant women regarding vaccine safety are a persistent barrier to vaccine acceptance during pregnancy. Previous studies of maternal COVID-19 vaccination and birth outcomes have been limited by small sample size (*2*) or lack of an unvaccinated comparison group (*3*). In this retrospective cohort study of live births from eight Vaccine Safety Datalink (VSD) health care organizations, risks for preterm birth (<37 weeks’ gestation) and small-for-gestational-age (SGA) at birth (birthweight <10th percentile for gestational age) after COVID-19 vaccination (receipt of ≥1 COVID-19 vaccine doses) during pregnancy were evaluated. Risks for preterm and SGA at birth among vaccinated and unvaccinated pregnant women were compared, accounting for time-dependent vaccine exposures and propensity to be vaccinated. Single-gestation pregnancies with estimated start or last menstrual period during May 17–October 24, 2020, were eligible for inclusion. Among 46,079 pregnant women with live births and gestational age available, 10,064 (21.8%) received ≥1 COVID-19 vaccine doses during pregnancy and during December 15, 2020–July 22, 2021; nearly all (9,892; 98.3%) were vaccinated during the second or third trimester. COVID-19 vaccination during pregnancy was not associated with preterm birth (adjusted hazard ratio [aHR] = 0.91; 95% CI = 0.82–1.01). Among 40,627 live births with birthweight available, COVID-19 vaccination in pregnancy was not associated with SGA at birth (aHR = 0.95; 95% CI = 0.87–1.03). Results consistently showed no increased risk when stratified by mRNA COVID-19 vaccine dose, or by second or third trimester vaccination, compared with risk among unvaccinated pregnant women. Because of the small number of first-trimester exposures, aHRs for first-trimester vaccination could not be calculated. These data add to the evidence supporting the safety of COVID-19 vaccination during pregnancy. To reduce the risk for severe COVID-19–associated illness, CDC recommends COVID-19 vaccination for women who are pregnant, recently pregnant (including those who are lactating), who are trying to become pregnant now, or who might become pregnant in the future (*4*).

VSD is a collaboration between CDC and nine health care organizations representing approximately 3% of the U.S. population. This observational retrospective study included singleton live births from eight VSD sites in California, Colorado, Minnesota, Oregon, Washington, and Wisconsin (Kaiser Permanente: Colorado, Northern California, Northwest, Southern California, and Washington; Denver Health; HealthPartners; and Marshfield Clinic). Females aged 16–49 years with estimated pregnancy start during May 17–October 24, 2020, and expected delivery dates, based on a 40-week gestation, during February 21–July 31, 2021, were included. This cohort was likely to be pregnant when COVID-19 vaccines were first authorized in the United States. Pregnancies ending in live birth were identified from standardized VSD files using a validated pregnancy algorithm. The algorithm uses *International Classification of Diseases, Tenth Revision, Clinical Modification* (ICD-10-CM) diagnosis codes, Current Procedural Terminology codes, birth records, and electronic health record data (last menstrual period and expected delivery date) to identify the date and gestational age for live births (*5*). The algorithm then estimates the pregnancy start date, equivalent to the last menstrual period. Receipt of COVID-19 vaccines was identified from standardized VSD files, incorporating electronic health record, claims, and state and regional immunization information system data. All COVID-19 vaccine doses administered from the last menstrual period through 3 days before delivery were included. Vaccines administered within 3 days of delivery were excluded to reduce potential misclassification of vaccines administered postpartum as having been administered during pregnancy.

Primary outcomes were preterm birth, defined as birth <37 weeks’ gestation, and SGA at birth, defined as birthweight <10th percentile for gestational age compared with a U.S. reference population (*6*). Gestational age was determined from the VSD pregnancy algorithm. Birthweight was ascertained from birth records or maternal-infant linked electronic health record data. Covariates were obtained from ICD-10-CM codes and administrative and electronic health record data. State-level percentages of positive COVID-19 test results during the second trimester were calculated using publicly available data.* Propensity to be vaccinated during pregnancy was estimated using a generalized additive model with binomial distribution and logit link, including calendar week of pregnancy start, maternal age, race/ethnicity, prenatal care adequacy, maternal comorbidities, neighborhood poverty, state-level percentage of positive COVID-19 test results during the second trimester, and VSD site. Time-dependent COVID-19 vaccine and COVID-19 diagnosis Cox models with standardized inverse probability weighting were used to estimate the aHR of any COVID-19 vaccination during pregnancy and preterm and SGA at birth outcomes. This approach accounts for immortal time bias because shorter-duration pregnancies or those ending in preterm birth provide less opportunity to be vaccinated during pregnancy (*7*). In addition, aHRs were calculated for receipt of a first or second dose of an mRNA vaccine and for vaccination in the second or third trimester. Analysis was performed using SAS software (version 9.4; SAS Institute). Associations are reported based on aHRs and 95% CIs. Statistical significance was defined as a p-value <0.05 with a two-sided test. This surveillance was approved by the institutional review boards of participating sites with a waiver of informed consent. This activity was reviewed by CDC and was conducted consistent with applicable federal law and CDC policy.^†^

A total of 55,671 potentially eligible pregnancies resulting in a live birth were identified in VSD. After excluding 67 females ineligible because of age (i.e., <16 or >49 years), 926 with multiple (e.g., twin or triplet) gestations, 2,489 with no documented care in the health system, 295 with implausible gestational age, and 5,815 with pregnancy start date outside the prespecified periods, 46,079 (82.8%) single-gestation pregnancies ending in live birth with data on gestational age remained. Among these, 10,064 pregnant women (21.8%) received ≥1 COVID-19 vaccine doses during pregnancy and during December 15, 2020–July 22, 2021. COVID-19 vaccination during pregnancy varied by maternal age, race/ethnicity, and selected maternal comorbidities (Table 1). First (or only) vaccine doses were received in the first trimester by 172 (1.7%) women, in the second trimester by 3,668 (36.5%), and in the third trimester by 6,224 (61.8%). Most women received mRNA vaccines, including 5,478 (54.4%) who received Pfizer-BioNTech and 4,162 (41.4%) who received Moderna vaccines; 424 (4.2%) received Janssen (Johnson & Johnson) vaccine. Among 9,640 women who received mRNA vaccines during pregnancy, 1,759 (18.2%) received 1 dose, and 7,881 (81.8%) received 2 doses (Table 2).

**TABLE 1:** Characteristics of women who received and did not receive COVID-19 vaccine during pregnancy,* by vaccination status — eight U.S. health care organizations,^†^ December 15, 2020–July 22, 2021

Characteristic	Vaccination status, no. (%)	
	Unvaccinated	Vaccinated
**All pregnancies with gestational age data**	36,015 (78.2)	10,064 (21.8)
**Pregnancy start date range (based on 2020 epidemiologic weeks)^§^**		
May 17–Jun 13	7,598 (21.1)	366 (3.6)
Jun 14–Jul 11	7,131 (19.8)	1,124 (11.2)
Jul 12–Aug 8	6,400 (17.8)	2,043 (20.3)
Aug 9–Sep 5	6,095 (16.9)	2,360 (23.5)
Sep 6–Oct 3	5,742 (15.9)	2,608 (25.9)
Oct 4–Oct 24	3,049 (8.5)	1,563 (15.5)
**Race/Ethnicity^¶^**		
Hispanic	13,840 (38.4)	2,462 (24.5)
White, non-Hispanic	11,588 (32.2)	4,325 (43.0)
Asian, non-Hispanic	5,642 (15.7)	2,571 (25.6)
Black, non-Hispanic	3,293 (9.1)	271 (2.7)
Other/Unknown**	1,652 (4.6)	435 (4.3)
**Prenatal care index^††^**		
Adequate/Plus	25,308 (70.3)	7,263 (72.2)
Intermediate	7,404 (20.5)	2,202 (21.9)
Inadequate	3,303 (9.2)	599 (5.9)
**Comorbidities** ^§§^		
Asthma	2,733 (7.6)	802 (8.0)
Cancer	120 (0.3)	28 (0.3)
Cardiovascular disease	104 (0.3)	43 (0.4)
COVID-19 disease^¶¶^	1,269 (3.5)	281 (2.8)
Diabetes (type I or II)	611 (1.7)	167 (1.7)
Hypertension	1,732 (4.8)	552 (5.5)
Liver disease	417 (1.2)	97 (1.0)
Obesity***	10,426 (29.0)	2,407 (23.9)
Smoking (ever)	7,242 (20.1)	1,786 (17.8)
Systemic lupus	103 (0.3)	20 (0.2)
**Age, mean (SD), yrs,**	29.8 (5.3)	32.3 (4.5)
**Percentage living in poverty, mean (SD)^†††^**	10.0 (8.0)	8.0 (7.0)
**State-level COVID-19 positivity, mean (SD)^§§§^**	9.0 (2.0)	9.0 (2.0)

* Vaccines administered during December 15, 2020–July 22, 2021; vaccinated refers to all COVID-19 vaccine doses (including first or second doses) administered from last menstrual period to 3 days before delivery.

^†^ Kaiser Permanente: Colorado, Northern California, Northwest, Southern California, and Washington; Denver Health (Colorado); HealthPartners (Minnesota); and Marshfield Clinic (Wisconsin).

^§^ The Vaccine Safety Datalink pregnancy algorithm was used to estimate the pregnancy start date (equivalent to the last menstrual period).

^¶^ Race/ethnicity came from electronic health data, based on self-report.

** Unknown refers to missing ethnicity and unknown race.

^††^ Adequacy of prenatal care defined based on the Kotelchuck prenatal care index.

^§§^ Presence of comorbidities defined as having one or more inpatient or two or more outpatient diagnoses for the period 3 years before pregnancy through 20 weeks’ gestation.

^¶¶^ COVID-19 disease during pregnancy based on having a COVID-19 diagnosis within 30 days before last menstrual period or during pregnancy.

*** Obesity was defined as having obesity diagnosis or body mass index ≥30 kg/m^2^ before pregnancy or during the first trimester.

^†††^ Percentage living in poverty by neighborhood (i.e., U.S. Census tract) from the American Community Survey 5-year summary for 2019.

^§§§^ Average state-level COVID-19 test positivity during second trimester was calculated using publicly available data (https://protect-public.hhs.gov/).

**TABLE 2:** Vaccination during pregnancy, by vaccine type, timing of first dose, and total doses received during pregnancy — eight U.S. health care organizations,* December 15, 2020–July 22, 2021

Characteristic	Vaccinated, no. (%)
**All**	**10,064 (100)**
**Vaccine type**	
Pfizer-BioNTech	5,478 (54.4)
Moderna	4,162 (41.4)
Janssen (Johnson & Johnson)	424 (4.2)
**Timing of first dose in pregnancy**	
First trimester	172 (1.7)
Second trimester	3,668 (36.5)
Third trimester	6,224 (61.8)
**Doses during pregnancy (mRNA vaccines)**	
At least 1	9,640 (100)
1 dose	1,759 (18.2)
2 doses	7,881 (81.8)

* Kaiser Permanente: Colorado, Northern California, Northwest, Southern California, and Washington; Denver Health (Colorado); HealthPartners (Minnesota); and Marshfield Clinic (Wisconsin).

The overall prevalence of preterm birth and SGA at birth were 6.6 and 8.2 per 100 live births, respectively (Table 3). COVID-19 vaccination during pregnancy was not significantly associated with increased risk for preterm birth overall (aHR = 0.91; 95% CI = 0.82–1.01; p = 0.06) or SGA at birth (aHR = 0.95; 95% CI = 0.87–1.03; p = 0.24), or when stratified by mRNA vaccine dose number during pregnancy, compared with the risk in unvaccinated pregnant women. There also was no association with increased risk for preterm or SGA at birth when evaluating vaccination by trimester for the first (or only) vaccine dose.

**TABLE 3:** Preterm births, small-for-gestational-age births, and adjusted hazard ratios* among women receiving COVID-19 vaccine during pregnancy compared with unvaccinated pregnant women — eight U.S. health care organizations,^†^ December 15, 2020–July 22, 2021

Event	No. of subjects	Prevalence (events per 100 live births)	aHR^§ ^(95% CI)
**Preterm birth** ^¶^			
**Full population**	46,079	6.6	NA
No COVID-19 vaccines during pregnancy	36,015	7.0	Ref
Any COVID-19 vaccine during pregnancy	10,064	4.9	0.91 (0.82–1.01)
mRNA vaccine, 1 dose	1,759	7.7	0.78 (0.66–0.93)
mRNA vaccine, 2 doses	7,881	4.3	0.97 (0.86–1.10)
Second trimester**	3,668	6.4	1.05 (0.90–1.23)
Third trimester**	6,224	4.0	0.82 (0.72–0.94)
**Small-for-gestational-age at birth** ^††^			
**Full population**	40,627	8.2	NA
No COVID-19 vaccines during pregnancy	31,699	8.2	Ref
Any COVID-19 vaccine during pregnancy	8,928	8.2	0.95 (0.87–1.03)
mRNA vaccine, 1 dose	1,576	8.2	0.92 (0.80–1.07)
mRNA vaccine, 2 doses	6,982	8.3	0.98 (0.89–1.08)
Second trimester**	3,226	8.6	1.00 (0.86–1.17)
Third trimester**	5,561	8.0	0.93 (0.85–1.02)

**Abbreviations:** aHR = adjusted hazard ratio; NA = not applicable; Ref = referent group.

* Associations were estimated using a time-dependent covariate Cox model with inverse probability weighting and COVID-19 disease status as a time-dependent covariate.

^†^ Kaiser Permanente: Colorado, Northern California, Northwest, Southern California, and Washington; Denver Health (Colorado); HealthPartners (Minnesota); and Marshfield Clinic (Wisconsin).

^§^ Inverse probability weighting was computed using a generalized additive model for receiving 1 or 2 doses of COVID-19 vaccines during pregnancy with calendar week of pregnancy start date, maternal age, race/ethnicity, prenatal care adequacy, maternal comorbidities, state level COVID-19 average test positivity during the second trimester, neighborhood poverty, and Vaccine Safety Datalink site as covariates.

^¶^ <37 weeks’ gestational age.

** Based on timing for first or only vaccine dose; first trimester vaccinations are not included in analyses stratified by trimester because few exposures occurred (172).

^††^ Birthweight for gestational age <10th percentile.

## Discussion

In this large, multisite, retrospective cohort study, receipt of COVID-19 vaccine during pregnancy was not associated with increased risk for preterm birth or SGA at birth. The absolute risk for severe morbidity associated with COVID-19 in pregnancy is low; however, women with symptomatic COVID-19 during pregnancy have a more than twofold increased risk for intensive care unit admission, invasive ventilation, and extracorporeal membrane oxygenation, and a 70% increased risk for death, compared with nonpregnant women with symptomatic infections (*8*). Evidence of the benefits of COVID-19 vaccination during pregnancy continues to accrue, including the detection of antibodies in cord blood (*9*). Together, these findings reinforce the importance of communicating the risks for COVID-19 during pregnancy, the benefits of vaccination, and information on the safety and effectiveness of COVID-19 vaccination during pregnancy.

To date, only a few reports have described outcomes among live births after COVID-19 vaccination in pregnancy. Data from CDC’s v-safe COVID-19 Vaccine Pregnancy Registry found that among live births, 9.4% were preterm, and 3.2% were SGA, consistent with background rates (*3*). The proportion identified as SGA at birth in the current study is higher than that in the v-safe registry likely because of variation in data sources (e.g., electronic health record data versus voluntary self-report) and calculation of SGA using different reference populations (U.S. versus international). An observational study from Israel also reported no association between COVID-19 vaccination in pregnancy and adverse maternal or birth outcomes (*2*). In addition, in a cohort study from the United Kingdom, among 1,328 pregnant women, 140 (10.5%) received a COVID-19 vaccine during pregnancy, and birth outcomes did not differ between vaccinated and unvaccinated women (*10*). The current study further demonstrates the safety of COVID-19 vaccination among pregnant women related to preterm birth and SGA at birth outcomes.

The findings in this report are subject to at least four limitations. First, although VSD sites access multiple data sources to identify receipt of COVID-19 vaccines during pregnancy, some vaccinations might have been missed, potentially biasing results toward the null. Second, data on selected confounders, such as previous history of preterm or SGA at birth, were not available, and data on previous infections with SARS-CoV-2 (the virus that causes COVID-19) that might have affected propensity to be vaccinated were not fully identified through previous COVID-19 diagnoses. In addition, reduced risks for preterm birth after third-trimester vaccination or receipt of a single mRNA vaccine dose during pregnancy were likely due to residual immortal time bias. Third, because of the timing of COVID-19 vaccine availability and the timing of the births in this cohort, few first-trimester vaccinations were observed. Nevertheless, the second and third trimester are critical periods for fetal growth and development. Risks associated with vaccination during the first trimester should be evaluated in future studies that include vaccines administered throughout pregnancy. Finally, this retrospective cohort does not include more recent pregnancies in women who might have been eligible for additional or booster vaccine doses during pregnancy.

Despite these limitations, the findings from this retrospective, multisite cohort of a large and diverse population with comprehensive data on vaccination, comorbidities, and birth outcomes add to the evidence supporting the safety of COVID-19 vaccination during pregnancy. CDC recommends COVID-19 vaccination for women who are pregnant, recently pregnant (including those who are lactating), who are trying to become pregnant now, or who might become pregnant in the future (4) to reduce the risk for severe COVID-19–associated outcomes.

**Summary**

**What is already known about this topic?**

Pregnant women with COVID-19 are at increased risk for severe illness and adverse birth outcomes, yet many remain reluctant to be vaccinated.

**What is added by this report?**

In a retrospective cohort of >40,000 pregnant women, COVID-19 vaccination during pregnancy was not associated with preterm birth or small-for-gestational-age at birth overall, stratified by trimester of vaccination, or number of vaccine doses received during pregnancy, compared with unvaccinated pregnant women.

**What are the implications for public health practice?**

These data support the safety of COVID-19 vaccination during pregnancy. CDC recommends COVID-19 vaccination for women who are pregnant, recently pregnant, who are trying to become pregnant now, or who might become pregnant in the future.

## Footnotes

*
https://protect-public.hhs.gov

† 45 C.F.R. part 46.102(l)(2), 21 C.F.R. part 56; 42 U.S.C. Sect. 241(d); 5 U.S.C. Sect. 552a; 44 U.S.C. Sect. 3501 et seq.
